# From Algorithms to Insight: The Transformative Power of Artificial Intelligence and Machine Learning in Urological Cancer Research

**DOI:** 10.3390/curroncol32050277

**Published:** 2025-05-14

**Authors:** Matthias May, Sabine Brookman-May, Emily Rinderknecht

**Affiliations:** 1 Department of Urology, St. Elisabeth Hospital Straubing, 94315 Straubing, Germany; 2 Department of Urology, LMU University Hospital, Ludwig Maximilian University of Munich, 81377 Munich, Germany; 3 Aura Biosciences, Boston, MA 02135, USA; 4 Department of Urology, St. Josef Medical Center, University of Regensburg, 93053 Regensburg, Germany

As we advance into a new era of oncological science, artificial intelligence (AI) is no longer a peripheral tool—it is a central agent of change. Nowhere is this transformation more profound and more urgently needed than in urologic oncology, a field that has emerged as one of the most dynamic and innovation-driven domains in contemporary cancer research [1]. Here, the imperatives of precision, personalization, and prognostication converge at unprecedented velocity. AI, in its various incarnations, from classical machine learning to cutting-edge multimodal foundation models, is not merely enhancing traditional methods; it is reshaping the epistemological foundations of cancer research.

Over the past decade, machine learning has matured from experimental prototypes to clinically embedded systems. Deep convolutional neural networks are now widely used to classify prostate lesions on multiparametric magnetic resonance imaging according to PI-RADS criteria, thereby reducing interobserver variability and improving the detection of clinically significant tumors [2,3,4]. More recent innovations, including Vision Transformers (ViTs) and hybrid models trained on multicenter data, offer refined differentiation between aggressive and indolent lesions—even in diagnostically ambiguous cases.

These are more than computational achievements; they represent cognitive accelerators that increase diagnostic confidence and shorten decision-making timelines [5].

The impact of AI in histopathology is equally transformative. Deep learning models trained on digitized whole-slide images now match or even exceed expert pathologists in Gleason grading accuracy [6,7], thereby offering a scalable solution to workforce limitations. Beyond automation, these systems unlock new frontiers: they can detect architectural and cytological features too subtle for the human eye, paving the way for novel digital biomarkers and redefined prognostic frameworks. In doing so, AI shifts histopathology from visual estimation to data-driven precision.

AI is also transforming the molecular landscape of urologic oncology. Techniques like unsupervised clustering and dimensionality reduction are now applied to high-dimensional transcriptomic and proteomic data, revealing novel therapeutic targets and resistance pathways. One of the most promising intersections is radiogenomics—where imaging features are linked with underlying molecular signatures [8]. This fusion of modalities, feasible only through AI, is already yielding results. In clear cell renal cell carcinoma, radiogenomic algorithms have accurately predicted VHL mutations and angiogenic profiles, enabling non-invasive molecular profiling with unprecedented precision [8,9].

Perhaps AI’s most transformative contribution lies in its power to integrate. Multimodal models now combine imaging, histopathology, molecular profiles, and electronic health data to create detailed individualized patient representations. These digital phenotypes go far beyond conventional endpoints, offering tailored predictions for recurrence, immune response, and quality of life. Large language models (LLMs) such as ChatGPT-4 and Claude 3.5 further extend this integration by streamlining and enhancing clinical workflows and decision-making and facilitating patient communication [10]. In recent studies, these models have produced patient-friendly summaries of urologic cancer trial information that match—or even surpass—those written by clinicians in terms of both clarity and empathy [11,12,13]. While more validation is needed across diverse patient populations, their potential to democratize oncology communication is clear.

This maturation of AI from proof of concept to clinical co-pilot is now producing a new generation of decision-support tools that extend beyond static prediction. By continuously integrating patient-specific variables, these systems support individualized treatment planning, real-time tumor board discussions, and nuanced risk stratification grounded in probabilistic reasoning [11,12,13,14,15]. Nevertheless, early randomized studies make it clear that technical sophistication alone does not guarantee improved outcomes. Without thoughtful integration into clinical workflows and adaptation by end-users, even the most accurate algorithms may fall short in practice [16]. The transition from retrospective validation to prospective, real-world implementation marks a fundamental shift—one that redefines the dynamic between algorithmic guidance and clinical judgment.

AI is also poised to reshape the architecture of experimental research itself. In silico hypothesis generation, AI-assisted trial recruitment, adaptive randomization frameworks, and truly adaptive trial designs may significantly reduce the inefficiencies that lead to high attrition and slow accrual. LLMs trained extensively on trial protocols and biomedical literature now support the conceptual design of randomized clinical trials (RCTs) by rapidly assessing endpoint validity, inclusion criteria, internal consistency, and statistical feasibility [17]. What once took weeks can now be accomplished in a fraction of the time. Looking ahead, the AI-enabled simulation of trial scenarios and outcomes across virtual cohorts could optimize eligibility criteria and estimate effect sizes based on real-world data, thereby enhancing both scientific precision and ethical responsibility. Beyond trial design, AI is also transforming operational layers of trial execution, including site selection and feasibility assessment, leveraging real-world data to identify high-performing centers. AI-driven models also forecast recruitment trajectories in real time, allowing dynamic adjustments such as targeted outreach or expanded site activation. Molecular pre-screening tools, integrated with EHR and genomic data, streamline trial matching and stratification. At the endpoint level, AI is helping validate novel digital biomarkers, thereby offering more sensitive and earlier readouts of treatment efficacy or toxicity. Finally, predictive algorithms can inform adaptive interim analyses and signal futility or success earlier than fixed schedules.

Federated learning introduces a new paradigm, enabling institutions to collaboratively train models on decentralized datasets without compromising patient confidentiality. This approach fosters the development of rare urologic cancer models (such as for patients with penile cancer) across international cohorts, maintaining stringent data privacy, a necessity given the biological and logistical heterogeneity that defines urologic oncology [18].

One of the most visionary innovations emerging at the intersection of AI and urologic cancer care is the concept of the digital twin: a dynamic, virtual model of an individual patient, built from real-time, multiscale data inputs. These digital avatars could revolutionize preclinical modeling by simulating therapeutic sequences, optimizing dosing strategies, and predicting long-term outcomes before any treatment is delivered [19]. Although still experimental, digital twins represent a shift toward a truly proactive, precision medicine. Their success will rely on advances in mechanistic modeling, systems biology, and improved interpretability of increasingly complex algorithmic architectures [20].

However, with great power comes great responsibility. AI’s potential can tempt us toward algorithmic overconfidence. Models trained on biased or incomplete data can perpetuate health disparities and reinforce systemic inequities. Regulatory frameworks are still catching up, struggling to match the pace of innovation. The opacity of “black box” systems, despite their accuracy, can erode clinical trust and accountability. As AI-generated manuscripts and synthetic datasets become more common, the need to safeguard scientific integrity and trust becomes a moral imperative. In this context, editorial stewardship gains renewed urgency and ethical weight. Journals like *Current Oncology* must not only spotlight innovation but also scrutinize it and favor studies that combine technical excellence with transparency, patient relevance, and ethical reflection. Peer review must evolve accordingly, becoming more multidisciplinary, ethically grounded, and data-literate.

The integration of AI into urologic oncology is not merely a technological evolution; it marks a profound philosophical inflection point. We are moving from empirical observation to predictive modeling, from retrospective analysis to anticipatory insight, and from human-exclusive interpretation to a new era of human–machine collaboration. This shift does not weaken clinical agency; it redefines and empowers it. As urologic oncologists, we remain the interpreters of meaning, even as AI reveals patterns and possibilities previously beyond our reach. AI is not the destination—it is a catalyst. Its promise lies not in replacing physicians but in uncovering hidden signals, latent structures, and new therapeutic frontiers. Our responsibility is to harness its capabilities with discernment, apply it to humility, and anchor its use in the ethical foundations that make medicine inherently human. The era of intelligent oncology is here. We must meet it not with hesitation but with clarity, courage, and a steadfast belief that human judgment must always lead.
